# Hydrogen Sulfide Promotes Cardiomyocyte Proliferation and Heart Regeneration *via* ROS Scavenging

**DOI:** 10.1155/2020/1412696

**Published:** 2020-05-21

**Authors:** Jianqiu Pei, Fang Wang, Shengqiang Pei, Ruifeng Bai, Xiangfeng Cong, Yu Nie, Xi Chen

**Affiliations:** State Key Laboratory of Cardiovascular Disease, Fuwai Hospital, National Center for Cardiovascular Disease, Chinese Academy of Medical Sciences and Peking Union Medical College, Beijing 100037, China

## Abstract

Neonatal mouse hearts can regenerate completely in 21 days after cardiac injury, providing an ideal model to exploring heart regenerative therapeutic targets. The oxidative damage by Reactive Oxygen Species (ROS) is one of the critical reasons for the cell cycle arrest of cardiomyocytes (CMs), which cause mouse hearts losing the capacity to regenerate in 7 days or shorter after birth. As an antioxidant, hydrogen sulfide (H_2_S) plays a protective role in a variety of diseases by scavenging ROS produced during the pathological processes. In this study, we found that blocking H_2_S synthesis by PAG (H_2_S synthase inhibitor) suspended heart regeneration and CM proliferation with ROS deposition increase after cardiac injury (myocardial infarction or apex resection) in 2-day-old mice. NaHS (a H_2_S donor) administration improved heart regeneration with CM proliferation and ROS elimination after myocardial infarction in 7-day-old mice. NaHS protected primary neonatal mouse CMs from H_2_O_2_-induced apoptosis and promoted CM proliferation *via* SOD2-dependent ROS scavenging. The oxidative DNA damage in CMs was reduced with the elimination of ROS by H_2_S. Our results demonstrated for the first time that H_2_S promotes heart regeneration and identified NaHS as a potent modulator for cardiac repair.

## 1. Introduction

Cardiovascular disease, the leading cause of death in humans, poses serious threats to life and is a heavy economic burden. Loss of cardiomyocyte (CM) induced by cardiac injury is the main cause of heart failure which is of awful prognosis [1]. Studies in recent decades have shown that lower vertebrate such as zebrafish maintains a heart-regenerating ability throughout their lives [2], while mammals, such as pig [3, 4], mouse [5], and even human [6, 7], have a transient ability to regenerate the heart when they were neonates. With postnatal development, mammals lose heart regeneration ability soon after birth. Mice lose this regenerative capacity by postnatal day (P)7 [5, 8], and pig can keep this potency just one day after birth [3, 4].

Recently, lineage tracing studies have found that newly generated CMs are mainly the result of division of preexisting CMs [9, 10]. For this reason, efforts have been made to identify the molecular mechanisms underlying postnatal cardiac cell cycle arrest. Researchers have found that the upstream signal triggering CMs to exit the proliferative cycle is related to reactive oxygen species (ROS) produced by oxidative metabolism [11, 12]. High levels of ROS are harmful to many processes; for example, they oxidize membrane lipids and amino acid residues of proteins, which may alter cell function and integrity [13]. ROS production associated with metabolism-induced DNA damage is a major cause of cell cycle arrest [14–16]. How to remove these metabolic byproducts safely and effectively is a key question in myocardial regeneration.

Hydrogen sulfide (H_2_S), like nitric oxide (NO) and carbon monoxide (CO), is an endogenous gas signaling molecule. After synthesis, H_2_S can spread into the environment surrounding cells or be stored in cells. In mammalian tissues, H_2_S is produced by both nonenzymatic and enzymatic catalysis, with cystathionine-*β*-synthase (CBS) and cystathionine-*γ*-lyase (CSE) enzyme catalysis being dominant [17]. H_2_S has been widely accepted to exhibit protective properties in many organs [18, 19], especially in the heart in the context of damage such as that from myocardial infarction (MI), ischemia-reperfusion (I/R), arrhythmia, cardiac hypertrophy, myocardial fibrosis, and heart failure [20, 21]. However, whether H_2_S plays an essential role during heart regeneration is still unknown.

We previously established neonatal mouse MI and apex resection (AR) models to study heart regeneration [22–25]. Employing the neonatal mouse heart regeneration model, here, we explored the role of H_2_S signaling in heart regeneration. The results showed that H_2_S improved CM cell cycle progression by scavenging ROS, thereby promoting heart regeneration. During this process, H_2_S-mediated ROS elimination was mainly SOD2-dependent.

## 2. Methods and Materials

### 2.1. Mice

The mice were provided by the National Center of Cardiovascular Disease. All experiments with mice were conducted according to the “Regulation to the Care and Use of Experimental Animals” of the Beijing Council on Animal Care (1996). The protocol was approved by the Fuwai Hospital Animal Care and Use Committee.

### 2.2. MI Model

The MI model was created in neonatal C57BL/6 mice (P2 or P7) through ligation of the left anterior descending coronary artery (LAD), as previously described [26]. Briefly, each mouse was anesthetized on ice for 2-4 min, and the chest was opened to expose the heart. The LAD in the left ventricle was ligated with 8-0 suture, and the chest was then closed with 8-0 nonabsorbable silk suture. The same procedure was performed for the sham-operated group except that the LAD was not ligated. Then, the mice were rewarmed under a heating lamp at 37°C for recovery. After surgery, cardiac function was assessed by echocardiography using a VisualSonics Vevo 2100 ultrasound system (VisualSonics, Inc.) At specific time points, the animals were sacrificed, and the hearts were dissected and processed for histological and other analyses.

### 2.3. Apex Resection

The neonatal mouse heart apex resection model was performed as described previously [24]. Briefly, neonatal C57BL/6J mice at postnatal day (P)2 were anesthetized by hypothermia and embedded in ice for 2 min. The skin was cut open with a micro scissors in the fourth intercostal space on the left side. The mouse's thorax was gently pressed, and the apex was pushed out; the apex was then resected using iridectomy scissors, and the chest and skin were sewn up with 8-0 nonabsorbable silk suture. For sham controls, the same procedures were performed without removing the heart apex. After surgery, mice were waked up under a heating pad.

### 2.4. ROS Measurement

The production of ROS was evaluated by analyzing the fluorescence intensity that resulted from dihydroethidium (DHE) (Invitrogen D11347) staining. In brief, frozen mouse hearts were cut into 5 *μ*m sections. Serial heart sections were stained with 5 *μ*M DHE at 37°C for 30 min and then measured by fluorescence microscopy (excitation at 490 nm, emission at 610 nm).

### 2.5. CM Isolation

CMs were isolated from the hearts of neonatal mice at P1 using a Neonatal Heart Dissociation Kit with a gentleMACS™ Octo Dissociator (Miltenyi Biotec, Teterow, Germany) according to the manufacturer's instructions and cultured in DMEM supplemented with 10% FBS at 37°C and 5% CO_2_.

### 2.6. In Vitro Oxidative Stress Induction

To induce oxidative stress injury, primary CMs (1.5 × 10^4^ per well) were differentiated in 96-well plates. The cells were treated with 500 *μ*M H_2_O_2_ in serum-free DMEM for 12 h in a 5% CO_2_ incubator at 37°C. H_2_O_2_ was obtained from AppliChem GmbH (Darmstadt, Germany). NaHS was purchased from Sigma-Aldrich Chemie GmbH (Taufkirchen, Germany).

### 2.7. Knockdown of Mn-SOD

Mn-SOD was knocked down with siRNA (Thermo AM16708). Ambion® Silencer® Negative Control #2 siRNA (Thermo AM 4613), which has no significant sequence similarity to mouse, rat, or human gene sequences, was used as the negative control. CMs were seeded into 12-well plates (1.0 × 10^6^ cells/well) for 24 h and then transfected with siRNA using Lipofectamine 3000 (Invitrogen, Waltham, USA) according to the manufacturer's instructions. The cells were collected 48 h after transfection.

### 2.8. Quantitative Real-Time PCR (qRT-PCR)

Total RNA was extracted from cells using TRIzol reagent and then quantified using a NanoDrop 2000 spectrophotometer. cDNA was generated from the total RNA (1 *μ*g) using M-MLV reverse transcriptase and oligo(dT)15 primers. qRT-PCR was performed using SYBR Green PCR Master Mix and an Applied Biosystems 7500 instrument (ABI, Foster City, CA, USA). The primer pairs used were as follows: SOD2—5′-CAGACCTGCCTTACGACTATGG-3′ (forward) and 5′-CTCGGTGGCGTTGAGATTGTT-3′ (reverse); *β*-actin—5′-AGCCATGTACGTAGCCATCC-3′ (forward) and 5′-CTCTCAGCTGTGGTGGTGAA-3′ (reverse).

### 2.9. Histology

The hearts were harvested, fixed in 4% paraformaldehyde at room temperature for 24 h, dehydrated in ethanol and xylene, and then embedded in paraffin. For the MI model, the hearts were longitudinally embedded and sectioned at 5 *μ*m thickness. With standard procedures, Masson's trichrome staining was performed. The thickness of the left ventricle anterior wall (LVAW) was calculated by Image-Pro Plus.

### 2.10. Immunostaining

Deparaffinization, antigen retrieval with 1 mM EDTA (pH 9.0) in boiling water, and blocking of nonspecific binding sites were performed. The sections were then incubated with primary antibodies overnight at 4°C, washed three times with PBS, and incubated with fluorescence-labeled secondary antibodies for 1 h at 25°C in the dark. The slides were washed three times in PBS, counterstained with DAPI (Sigma-Aldrich, St. Louis, MO, USA), and mounted with VECTASHIELD (Vector Labs, CA, USA). The primary antibodies used were as follows: anti-phospho Histone H3 Ser10 (Millipore #06-570, 1 : 100), anti-Ki67 (Abcam, ab16667, 1 : 200), anti-Aurora B (1 : 100; ab2254, Abcam), and anti-Sarcomeric Alpha Actinin (Abcam, ab9465, 1 : 500). The anti-rabbit Alexa Fluor 488-conjugated (1 : 500; A-21206) and anti-mouse Alexa Fluor 594-conjugated (1 : 500; A-21203) secondary antibodies were from Invitrogen. Fluorescence was observed under a ZEISS LSM800 confocal laser scanning microscope (Carl Zeiss, Inc., Jena, Germany).

### 2.11. Western Blot Analysis

CMs were lysed in a RIPA buffer that contained protease inhibitors (Roche, Basel, Switzerland). After centrifugation (15000 × *g*, 10 min, 4°C), the cell lysate protein concentrations were determined by BCA Protein Assay (Beyotime Institute of Biotechnology, Beijing, China). The membranes were blotted with the indicated antibodies. Some membranes were stripped and reblotted with an actin antibody. Monoclonal primary antibodies against phosphorylated-Ataxia Telangiectasis Mutated (pATM) (1 : 1000; Santa Cruz sc-47739), Mn-SOD (1 : 1000; Millipore Millipore-06-984), phosphorylated checkpoint kinase 1 (p-Chk1) (Ser296) (1 : 1000; CST #90178), phosphorylated checkpoint kinase 2 (p-Chk2) (Thr68) (1 : 1000; CST #2197), and GAPDH (1 : 5000; Sigma G9545) were used.

## 3. Results

### 3.1. Inhibition of H_2_S Impairs Mouse Neonatal Heart Regeneration

**Figure 1:**
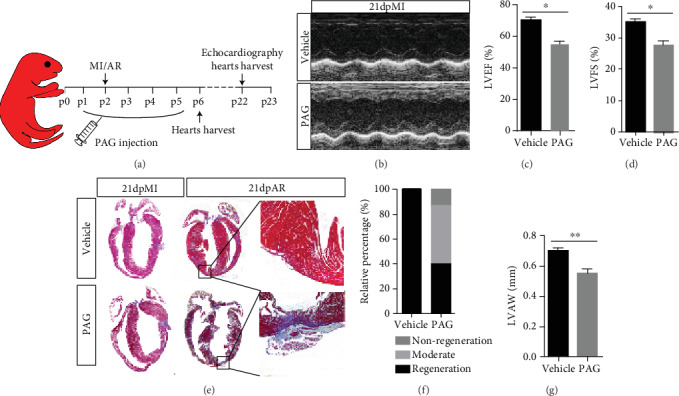
Inhibition of H_2_S synthase with PAG impairs heart regeneration. (a) Schema of the animal experiment. (b–d) Representative images and statistics of echo analysis 21 days post MI. LVEF: left ventricular ejection fraction; LVFS: left ventricular fractional shortening. (e) Representative images of Masson's trichrome-stained heart sections from mice 21 days post MI or AR. (f) Statistics of neonatal mouse heart regeneration. (g) LVAWd: left ventricular anterior wall diastolic thickness. Vehicle: *n* = 6; PAG: *n* = 15. The data are presented as the mean ± SEM. ^∗^*p* < 0.05 and ^∗∗^*p* < 0.01 by Student's *t*-test.

Endogenous H_2_S is derived from the catalytic activity of two enzymes: CBS, which is particularly expressed in the central nervous system, and CSE, which is primarily in the cardiovascular system. Therefore, to determine the functional significance of H_2_S signaling in heart regeneration, CSE in neonatal mice was inhibited with propargylglycine (PAG). The mice were subjected to permanent LAD ligation or AR at P2, the time point associated with a strong heart regenerative capability. The experimental schedule is shown in Figure 1(a). After 21 days, echocardiography revealed that heart function was significantly deteriorated in the PAG-treated group compared with the vehicle-treated group (Figures 1(b)–1(d)). Additionally, the hearts of the vehicle group were completely regenerated with little scarring, while those of the PAG-treated group showed large fibrotic scars (Figure 1(e)) and suppressed regenerative ability (Figure 1(f)).

### 3.2. PAG Reduces Proliferative Capability of Cardiomyocytes in Neonatal Mice

**Figure 2:**
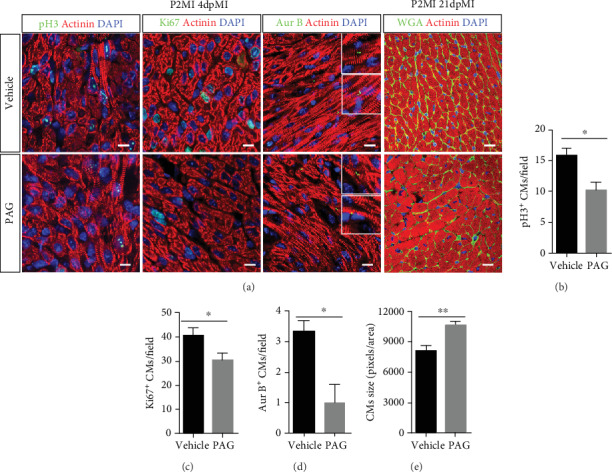
Inhibition of H_2_S synthase with PAG impedes CM proliferation. (a–d) Representative images and related statistical results of CM mitosis and cytokinesis, as analyzed by pH3, Ki67, and Aurora B staining. Actinin was used to label CMs, and DAPI was used to label nuclei. Scale bar =50 *μ*m. Vehicle: *n* = 4; PAG: *n* = 5. (a, e) Cell size was measured by WGA staining. Actinin was used to label CMs, and DAPI was used to label nuclei. Scale bar = 20 *μ*m. Vehicle: *n* = 3; PAG: *n* = 5. The data are presented as the mean ± SEM. ^∗^*p* < 0.05 and ^∗∗^*p* < 0.01 by Student's *t*-test.

To determine whether PAG treatment impeded CM proliferation during neonatal heart regeneration, we examined CM proliferation with the mitosis markers pH3 and Ki67, and cytokinesis marker Aurora B, at 4 days postsurgery. PAG treatment decreased the numbers of proliferative myocytes in the injured myocardium (Figures 2(a)–2(d)). Wheat germ agglutinin (WGA) staining for cell size assessment with ImageJ revealed a significantly decreased CM size in PAG-treated mouse hearts compared with vehicle-treated mouse hearts (Figures 2(a) and 2(e)), suggesting that inhibition of H_2_S signaling impairs CM proliferative capability in neonatal mouse hearts following injury.

### 3.3. NaHS Promotes Heart Regeneration and CM Proliferation

**Figure 3:**
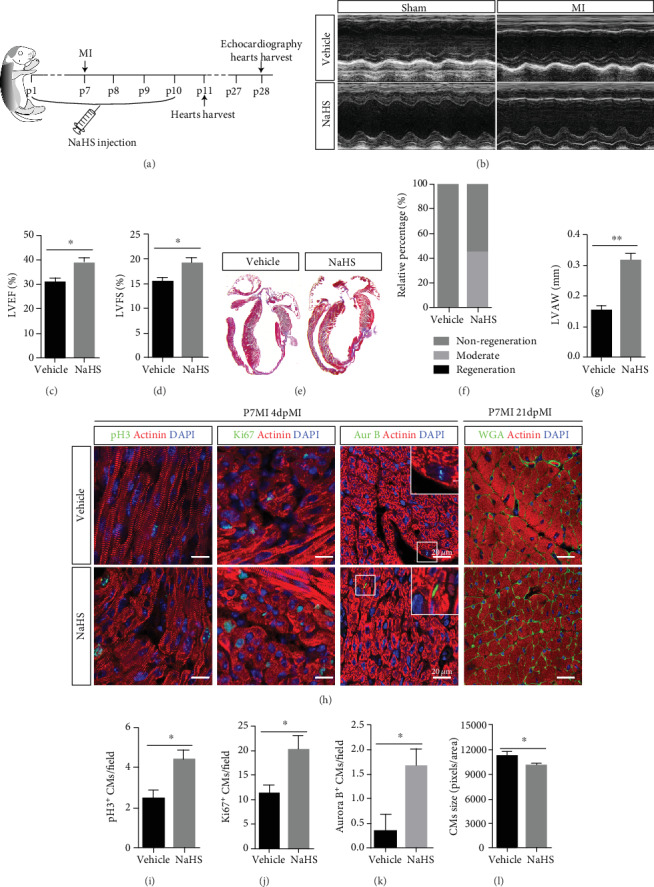
NaHS promotes heart regeneration related to CM proliferation. (a) Schema of the animal experiment. (b–d) Representative images and statistics of echo analysis 21 days post MI. LVEF: left ventricular ejection fraction; LVFS: left ventricular fraction shortening. (e) Representative images of Masson's trichrome-stained heart sections from mice 21 days post MI. (f) Neonatal mouse heart regeneration percentages. (g) LVAWd: left ventricular anterior wall diastolic thickness. Vehicle: *n* = 8; NaHS: *n* = 15. (h–k) Representative images and related statistical results of CM mitosis and cytokinesis, as indicated by pH3, Ki67, and Aurora B staining. Actinin was used to label CMs, and DAPI was used to label nuclei. Vehicle: *n* = 4; PAG: *n* = 5. Scale bar = 50 *μ*m. (l) Cell size was measured by WGA staining. Actinin was used to label CMs, and DAPI was used to label nuclei. Scale bar = 20 *μ*m. Vehicle: *n* = 3; PAG: *n* = 5. The data are presented as the mean ± SEM. ^∗^*p* < 0.05 and ^∗∗^*p* < 0.01 by Student's *t*-test.

Although mice exhibit a strong heart regenerative capability during the neonatal stage, the window closes at P7 [5, 8, 27]. To further investigate the effect of H_2_S on heart regeneration, we treated neonatal mice with NaHS (a donor of H_2_S) daily for 10 days after birth and subject them to LAD ligation at P7 (Figure 3(a)). The mice underwent echocardiographic measurement of left ventricular function 21 days after cardiac injury (Figures 3(b)–3(d)). Masson trichrome staining showed that the NaHS-treated hearts had smaller scars, less fibrosis with collagen deposition (Figure 3(e)), and enhanced regeneration (Figure 3(f)) compared with the vehicle group. There was a significant improvement in the LVAW thickness in the NaHS-treated group (Figure 3(g)), which implies that NaHS does have a positive role in heart regeneration. To confirm this, we examined CM proliferation in NaHS-treated heart tissues. Immunofluorescence staining for pH3, Ki67, and Aurora B revealed that there were more proliferative CMs in NaHS-treated hearts than in vehicle hearts (Figures 3(h)–3(k)). WGA staining for cell size assessment revealed that CMs were smaller in NaHS-treated mouse hearts than in vehicle mouse hearts (Figures 3(h) and 3(l)). Taken together, these results indicate that H_2_S promotes heart regeneration and improves heart function after MI in P7 mice by enhancing CM proliferation.

### 3.4. H_2_S Mitigates DNA Damage-Mediated Cell Cycle Arrest

**Figure 4:**
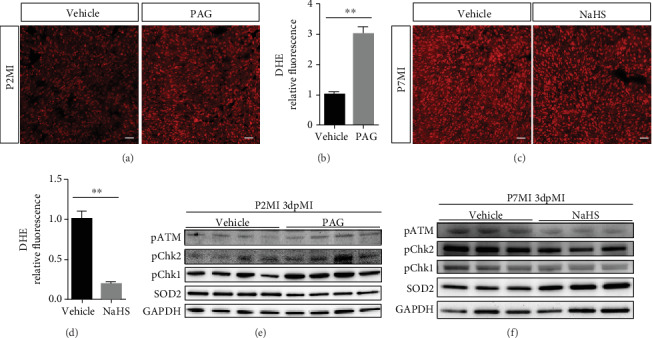
H_2_S is correlated with ROS scavenging and DNA damage during heart regeneration. (a–d) ROS levels in hearts after injury. (a, c) Representative images of DHE-stained heart sections from mice 1 day post MI. Scale bar = 20 *μ*m. (b, d) Relative index of DHE fluorescence. *n* = 3 per group. (e) DNA damage during oxidative stress was detected with western blotting (WB) in PAG-treated mouse hearts 3 days after MI. (f) DNA damage during oxidative stress was detected with WB in NaHS-treated mouse hearts 3 days after MI. The data are presented as the mean ± SEM. ^∗∗^*p* < 0.01 by Student's *t*-test.

To determine whether ROS scavenging is involved in H_2_S-mediated heart regeneration, we examined ROS deposition levels in frozen sections using DHE staining. We found that there were significantly greater ROS levels in PAG-treated mouse hearts than in vehicle-treated mouse hearts 3 days post MI (Figures 4(a) and 4(c)). In contrast, the ROS levels were lower in NaHS-treated mouse hearts (Figures 4(b) and 4(d)). These findings indicate that H_2_S-mediated promotion of CM proliferation during heart regeneration may be correlated with ROS scavenging.

ROS deposition is a major cause of oxidative DNA damage. Once a cell suffers DNA damage, the cell cycle checkpoint is activated, causing the cell to become arrested in the G1 or G2 phase [28, 29]. Here, we assessed the expression of pATM, Chk1, and Chk2, important markers of the DNA damage response. The results showed increased expression of pATM, p-Chk1, and p-Chk2 in PAG-treated mice but decreased expression of these proteins in NaHS-treated mice compared with vehicle-treated mice. Consistent with these findings, a crucial antioxidant enzyme, Mn-SOD (SOD2), was downregulated in the PAG-treated group but upregulated in the NaHS group compared with the vehicle-treated group (Figures 4(e) and 4(f)). These results reveal that H_2_S signaling can attenuate the DNA damage response during cell cycle progression.

### 3.5. H_2_S Suspends the ROS-Caused Cell Cycle Arrest in Primary CM

**Figure 5:**
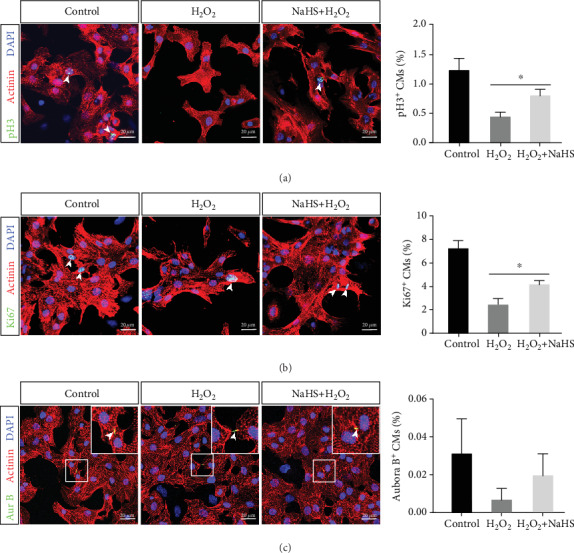
H_2_S exerts proliferative effects on CMs in vitro under conditions of H_2_O_2_ stimulation. Primary CMs from neonatal mice were cultured under H_2_O_2_ stimulation. (a, b) Representative images of pH3 and Ki67 immunofluorescence staining. And quantification of CM proliferation, as indicated by pH3 and Ki67 positive staining. (c) Representative images of Aurora B immunofluorescence staining and statistical results. Actinin was used to label CMs, and DAPI was used to label nuclei. Scale bar = 20 *μ*m. The data are presented as the mean ± SEM. ^∗^*p* < 0.05 by Student's *t*-test.

H_2_O_2_ treatment reduced the proliferative capacity of CMs, as quantified by pH3 and Ki67 immunohistochemistry assays, while NaHS treatment attenuated this reduction (Figures 5(a) and 5(b)). In addition, Aurora B staining showed a tendency of proliferation promotion, although not significant (Figure 5(c)). We also studied the protective effect of H_2_S on cardiomyocytes under oxidative stress (Supplement Figure 1). Overall, these findings imply that H_2_S signaling can reduce barriers to CM proliferation.

### 3.6. SOD2 Is Required for H_2_S-Mediated CM Proliferation

**Figure 6:**
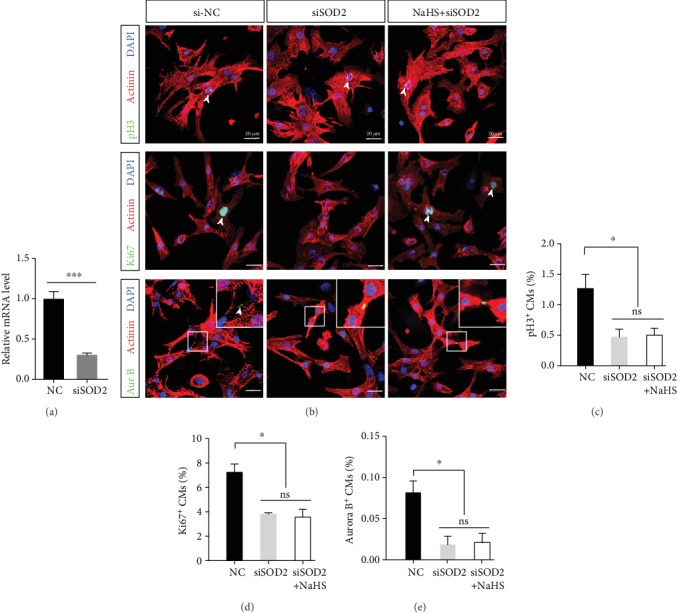
SOD2 is crucial to CM proliferation under physiological conditions. Primary CMs from P1 mice were treated with siSOD2 and NaHS. (a) Knockdown efficiency of siSOD2. ^∗∗∗^*p* < 0.001 by Student's *t*-test. (b) Representative images of pH3 and Ki67 immunofluorescence staining. Actinin was used to label CMs, and DAPI was used to label nuclei. Scale bar = 20 *μ*m. (c–e) Quantification of CM proliferation, as indicated by pH3, Ki67, and Aurora B positive staining. The data are presented as the mean ± SEM. ^∗^*p* < 0.05; ns: not significant, by one-way ANOVA with Bonferroni's multiple comparison test.

In mitochondria, a variety of antioxidant enzymes are important in determining ROS levels and maintaining cardiac function [30, 31]. Among them, SOD2 plays a critical role in ROS scavenging. Previous results have shown that PAG treatment reduces the expression of SOD2. To explore whether H_2_S-induced CM proliferation is mediated by SOD2, we knocked down SOD2 in primary CMs with siRNA. qRT-PCR revealed that SOD2 siRNA dramatically suppressed the expression of SOD2 (Figure 6(a)). Immunostaining revealed that downregulation of SOD2 impaired CM proliferation. Under siSOD2 treatment, CM proliferation was not greater in the H_2_S group than in the control group, as indicated by pH3, Ki67, and Aurora B immunofluorescence staining (Figures 6(b)-6(e)). These results indicate that SOD2 is required for H_2_S-mediated CM proliferation.

## 4. Discussion

In this study, we demonstrated that H_2_S signaling exerts a protective effect in the heart and plays a role in maintaining CM proliferation and heart regeneration after injury, with neonatal mouse heart regeneration AR and MI models. Inhibition of the H_2_S synthase CSE with PAG caused structural and functional defects in neonatal mouse hearts with decreased CM proliferation. In contrast, treatment with NaHS, a donor of H_2_S, promoted heart repair, increasing CM proliferation and decreasing ROS deposition and fibrosis. H_2_O_2_-mediated CM injury was mitigated by NaHS, and NaHS treatment improved CM proliferation capacity by attenuating ROS-induced cellular DNA damage, which may cause cell cycle arrest.

H_2_S regulates a variety of cellular signals and is involved in the regulation of cell death, differentiation, and proliferation [19]. It has been widely accepted that H_2_S is not only a secondary reaction product but also a critical mediator of the pathophysiological processes of many diseases. Over the past few years, a broad range of studies has shown that H_2_S plays important roles in renal ischemic injury repair [32] and renal fibrosis alleviation [33], lung disease repair [34], burn healing [35], and bone damage repair and bone regeneration [19]. In particular, the effects of H_2_S in cardiac ischemia injury repair and function preservation have been well studied.

Inhibition of CSE with PAG has been shown to increase infarct size in an *ex vivo* I/R study [36]. Confirming this finding, CSE knockout aggravates heart damage after I/R in mice [37]. Conversely, H_2_S produced endogenously through cardiac-specific overexpression of CSE significantly limits the extent of injury after MI [38]. All of the above findings have shown that H_2_S signaling has protective effects on adult mouse and rat hearts. In accord with these reports, our results showed that H_2_S signaling could promote heart regeneration and preserve heart function, further demonstrating the protective role of H_2_S in a neonatal mouse heart.

Immediate reperfusion of the occluded coronary artery is the gold standard method for treating MI and reducing associated mortality. However, re-recovery of blood flow in the ischemic myocardium often leads to the loss of function or even the death of myocardial cells, causing cardiac reperfusion injury [39]. A large number of mechanistic studies have shown that this damage may be related to intracellular calcium overload and the formation of ROS [40, 41]. ROS can cause lipid peroxidation, leading to destruction of the cell membrane structure and thus causing cell swelling. Many studies have confirmed that blocking the production of or scavenging oxygen free radicals such as ROS can improve cardiac function after cardiac I/R and reduce myocardial damage [42, 43].

Mammalian CMs, as terminally differentiated cells, lose their proliferative capacity soon after birth. Studies have found that mammals, such as mice, are in a hyperoxic environment after birth, and their metabolic patterns change from anaerobic glycolysis to mitochondrial oxidative phosphorylation [11]. The electron leakage of the electron transport chain during oxidative phosphorylation causes ROS production and accumulation, and ROS accumulation gradually increases with growth and development [44]. Excessive ROS can cause DNA damage, leading to the cell cycle arrest of CMs; this arrest causes CMs to exit the proliferative cycle, making the mammalian heart nonrenewable after injury. Consistent with the above results, we found that H_2_S can clear ROS, thereby promoting the reentry of CMs into the proliferative cycle.

H_2_S, a major antioxidant in mammalian tissues and cells, is usually present at low concentrations ranging from 10 to 30 nM [45–47]; however, its concentrations are 20- to 100-fold higher in the heart and aorta [48], implying a prominent regulatory role in the cardiovascular system. Studies have reported that H_2_S is involved in myocardial protection during I/R injury and that this protective effect is mainly derived from the antioxidative, anti-inflammatory, and antiapoptotic properties of H_2_S [49–51]. H_2_S can be used directly as an antioxidant to scavenge superoxide anions such as ROS. Many kinds of antioxidant enzymes can scavenge superoxide anions, and SOD is one of the most important candidates. Of the three types of SOD, SOD2 is mainly located in the mitochondria, which is consistent with the location of ROS produced from oxidative respiration. It has been reported that NaHS treatment can increase the expression of SOD2 and improve the activity of SOD1 in kidney tissues [32]. This study also found that NaHS treatment can increase the expression of SOD2 in the myocardium in neonatal mice and that SOD2 plays a role in ROS clearance and promotes CM proliferation. Furthermore, we found that H_2_S signaling-mediated ROS clearance reduces myocardial cell size. This finding is consistent with a previous study by Luo et al. showing that angiotensin II treatment increases ROS accumulation in myocardial tissue, which can cause hypertrophy of CMs [52].

## 5. Conclusions

In this study, we have shown, for the first time, that NaHS, an H_2_S donor, has the ability to promote heart regeneration by scavenging ROS to reduce ROS-induced DNA damage, thus promoting CM reentry into the cell cycle. This study revealed the potential benefits of NaHS in heart regeneration, and identifying H_2_S may be a potential target for myocardial injury therapy in clinical applications.

## Data Availability

All data used to support the findings of this study are included within the article. Raw data used to generate the figures are available from the corresponding author upon request.

## Supplementary Materials

Supplementary Materials Supplement Figure 1: H_2_S exerts protective effects on CMs. Primary CMs from neonatal mice were cultured under H_2_O_2_ stimulation. (A) Apoptosis was tested by Hoechst 33258 staining. Scale bar = 50 *μ*m. (B) Cell viability was measured by CCK-8 assay. (C) Representative images of TUNEL staining from CM and related statistical results. (D) Flow cytometric analysis of apoptosis in CMs subjected to H_2_O_2_ and quantification of CM apoptosis by Annexin V and PI flow cytometry assay. (E) Representative images of TUNEL immunofluorescence staining from heart. Actinin was used to label CMs, and DAPI was used to label nuclei. Vehicle: *n* = 5, NaHS: *n* = 5. Scale bar =50 *μ*m. The data are presented as the mean ± SEM; ns: not significant. ^∗^*p* < 0.05 by Student's *t*-test.
