# Supplementary material for: Hydrogen Sulfide Promotes Cardiomyocyte Proliferation and Heart Regeneration via ROS Scavenging
Source: Oxid Med Cell Longev. 2020 May 21;2020:1412696. doi: 10.1155/2020/1412696 (PMC7261318; doi:10.1155/2020/1412696)
Supplement: Supplementary Materials — Supplement Figure 1: H2S exerts protective effects on CMs. Primary CMs from neonatal mice were cultured under H2O2 stimulation. (A) Apoptosis was tested by Hoechst 33258 staining. Scale bar = 50 μm. (B) Cell viability was measured by CCK-8 assay. (C) Representative images of TUNEL staining from CM and related statistical results. (D) Flow cytometric analysis of apoptosis in CMs subjected to H2O2 and quantification of CM apoptosis by Annexin V and PI flow cytometry assay. (E) Representative images of TUNEL immunofluorescence staining from heart. Actinin was used to label CMs, and DAPI was used to label nuclei. Vehicle: n = 5, NaHS: n = 5. Scale bar =50 μm. The data are presented as the mean ± SEM; ns: not significant. ∗p < 0.05 by Student's t-test. [file 1412696.f1.pdf]

Hydrogen Sulfide Promotes Cardiomyocyte Proliferation and Heart Regeneration *via* ROS Scavenging

Supplemental Material

Supplemental Methods

Cell viability assessment: Proliferation assays were performed with a Cell Counting Kit-8 (CCK-8; Dojindo Laboratories, Rockville, Maryland) according to the manufacturer's protocol. This assay enables the determination of cell viability in proliferating cells though measurement of dehydrogenase activity, which is directly proportional to the number of living cells. Briefly, the cells were plated in a 96-well flat-bottom plate. After the cells were completely attached to the bottom, the cells were starved in medium without FBS for 16-18 h. Then, the medium was replaced with a medium: CCK-8 reagent mixture (10:1 by volume). Four hours later, the absorbance at 450 nm was measured as the baseline with an Automatic Enzyme Label Analyzer (Tecan Infinite M200 PRO). Then, the cells were incubated with the indicated reagents for 48 h. Finally, the culture medium was again replaced with the medium: CCK-8 reagent mixture. After 4 h, the absorbance at 450 nm was measured to show cell viability.

Cell apoptosis analysis

Hoechst 33258 staining and Flow cytometry assay: Cells after stimulated, aspirate the culture medium, add 0.5 ml of fixing solution, fix for 10 minutes, wash twice with PBS for 3 minutes, then adding 0.5 ml of Hoechst 33258 staining solution, stain for 5 minutes, and remove the staining solution. Wash twice with PBS for 3 minutes each time. Aspirate the liquid, and mount the slide with anti-fluorescence quenching mounting solution. Observed under a fluorescent microscope, the nuclei of apoptotic cells are densely stained, or fragmented. Or after incubation, cell apoptosis was evaluated by flow cytometry assay after Annexin V/propidium iodide (PI) (BD 556547 Anxn V FITC Apoptosis DETC KIT) staining according to the operating instruction.

TUNEL assay: Apoptasis assay was performed with TUNEL assay Kit (Roche) according to the manufacturer's protocol. Antigen retrieval with Citric acid (pH 6.0) in boiling water for 1 minute, then covered by 0.3% Triton X-100 dissolved in Citric acid (pH 6.0) for 8 minutes. After that, blocking of nonspecific binding sites was performed. The slides were then incubated with anti-Actinin overnight at 4 ℃, washed three times with PBS, and incubated with fluorescence-labeled secondary antibodies for 1 h at 25 ℃ in the dark. Then slides were incubated with TUNEL for 30 minutes at 37 ℃. Finally, the slides were mounted with DAPI (Sigma-Aldrich, St. Louis, MO, USA).

Supplementary Figure

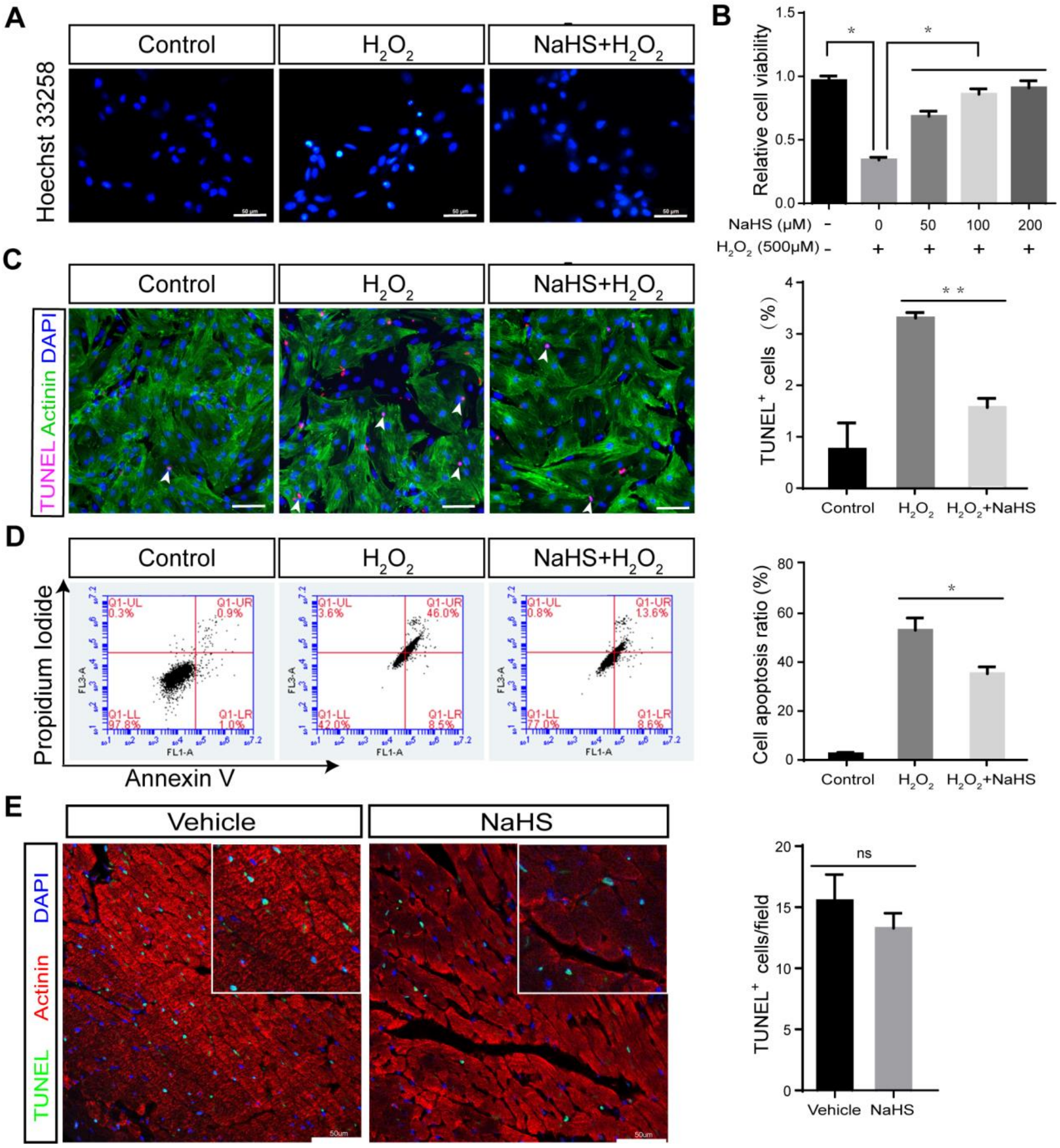

Supplement Figure: H_2S exerts protective effects on CMs. Primary CMs from neonatal mice were cultured under H_2O_2 stimulation. (A) Apoptosis was tested by Hoechst 33258 staining. Scale bar=50 μm. (B) Cell viability was measured by CCK-8 assay. (C) Representative images of TUNEL staining from CM and related statistical results. (D) Flow cytometric analysis of apoptosis in CMs subjected to H_2O_2, and quantification of CM apoptosis by Annexin V and PI flow cytometry assay. (E) Representative images of TUNEL immunofluorescence staining, Actinin was used to label CMs, and DAPI was used to label nuclei. (Vehicle n=5, NaHS n=5) Scale bar=50 μm. The data are presented as the mean ± SEM, ns not significant $*p<0.05$, $**p<0.01$ by Student's t-test.
